# Avaliação da Congestão Pulmonar por Ultrassom e Sensoriamento Dielétrico Remoto (ReDS) em Pacientes Hospitalizados com Insuficiência Cardíaca

**DOI:** 10.36660/abc.20240128

**Published:** 2024-10-23

**Authors:** Zhanna Kobalava, Ayten Fuadovna Safarova, Veronika Tolkacheva, Flora Elisa Cabello-Montoya, Olga Tairovna Zorya, Ivan Sergeevich Nazarov, Artem Alekseevich Lapshin, Ilya Pavlovich Smirnov, Nutsiko Ivanovna Khutsishvili, Maria Vatsik-Gorodetskaya

**Affiliations:** 1 RUDN University Moskva Moskovskaa Oblast Rússia RUDN University, Moskva, Moskovskaa Oblast – Rússia

**Keywords:** Insuficiência Cardíaca, Edema Pulmonar, Ultrassonografia

## Abstract

**Fundamento:**

A redução da congestão pulmonar é um alvo clínico essencial no tratamento da insuficiência cardíaca crônica (ICC). O sistema de sensoriamento dielétrico remoto ( *Remote Dielectric Sensing* ReDS) é uma tecnologia não invasiva recentemente introduzida, usada para estimar facilmente o grau de volume de fluido pulmonar sem nenhuma técnica especializada.

**Objetivo:**

Realizar uma avaliação comparativa da congestão pulmonar de acordo com a tecnologia de ultrassom e ReDS em pacientes hospitalizados com descompensação de ICC.

**Métodos:**

O estudo piloto de centro único incluiu pacientes hospitalizados com descompensação de ICC. Na admissão e na alta, a ultrassonografia pulmonar e a tecnologia ReDS foram realizadas simultaneamente. A ultrassonografia dos pulmões foi realizada de acordo com o protocolo com uma avaliação de 8 zonas e cálculo da soma das linhas B. A congestão pulmonar foi confirmada com uma soma de linhas B ≥5, congestão ReDS se >35%. Um p<0,05 foi considerado estatisticamente significativo.

**Resultados:**

Foram incluídos no estudo 35 pacientes; 40% (n=14) eram homens, a média de idade foi de 71 (65,5; 78,5) anos. A congestão pulmonar, segundo a ultrassonografia, foi de 57,1% (n=20), e segundo o ReDS, 62,9% (n=22). Foi encontrada correlação moderada entre o ReDS (%) e a ultrassonografia pulmonar (soma das linhas B) na admissão (coeficiente de correlação de Spearman = 0,402; p=0,017). Não houve correlação entre os dois métodos na alta (p=0,613).

**Conclusão:**

Houve correlação moderada entre ReDS e ultrassonografia pulmonar em relação à detecção de congestão pulmonar na admissão.

## Graphical abstract

**Figura Central:**
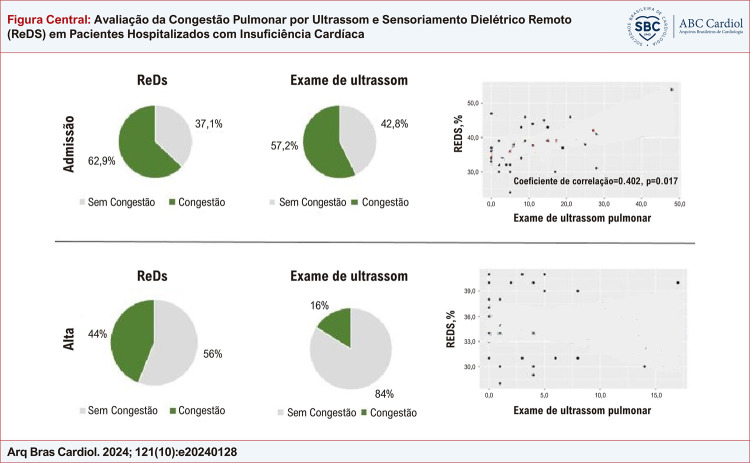
: Avaliação da Congestão Pulmonar por Ultrassom e Sensoriamento Dielétrico Remoto (ReDS) em Pacientes Hospitalizados com Insuficiência Cardíaca

## Introdução

A descompensação aguda da insuficiência cardíaca (ICAD) é baseada em uma cascata multinível de reações patológicas, que incluem sobrecarga hemodinâmica e congestão venosa. Avaliar o estado volêmico ou o estado de hidratação e, se possível, quantificar o grau de congestão é uma das tarefas mais importantes na estratégia de gerenciamento de pacientes em ambientes de internação e ambulatorial.

A congestão residual na alta é um problema sério associado a um risco aumentado de re-hospitalização e mortalidade.^1 - 3^ Além disso, pacientes sem manifestações clínicas de congestão podem apresentar congestão subclínica na alta, que é detectada apenas por métodos laboratoriais e instrumentais, e as manifestações clínicas podem se desenvolver mesmo antes do final da primeira semana de observação do paciente em regime ambulatorial.^2 , 3^

Mais de 90% das hospitalizações relacionadas à IC ocorrem devido à presença de congestão pulmonar (ensaio CHAMPION).^4^ A detecção precoce da congestão pulmonar é extremamente importante, pois permite prevenir o desenvolvimento de um episódio de descompensação a tempo, corrigir o tratamento a tempo e melhorar o prognóstico.

Atualmente, recomenda-se o uso de radiografia de tórax, ultrassonografia pulmonar, ecocardiografia e/ou peptídeos natriuréticos para detectar congestão em pacientes com IC aguda.^5 , 6^ Ao analisar dados de grandes registros estrangeiros e ensaios clínicos randomizados, onde a ultrassonografia pulmonar e a radiografia foram comparadas para o diagnóstico de síndrome intersticial cardiogênica em pacientes com IC, verificou-se que a ultrassonografia pulmonar não é apenas um método mais sensível para avaliar a congestão pulmonar, mas também tem valor prognóstico independente.^7 - 9^

No entanto, nenhum dos métodos permite avaliar o grau de sobrecarga de fluidos com precisão e, portanto, continua sendo necessário encontrar uma tecnologia nova, precisa e simples para avaliar a congestão nos pulmões. A urgência desse problema levou ao desenvolvimento de uma nova tecnologia não invasiva de Sensoriamento Dielétrico Remoto (ReDS), que é um método quantitativo validado para medir o volume total de fluido nos pulmões, determinando as propriedades dielétricas do tecido. O uso dessa tecnologia permite medir de forma rápida, não invasiva e quantitativa o conteúdo de fluido nos pulmões, permite otimizar o regime de tratamento e reduz o número de hospitalizações repetidas.^10^ No entanto, de acordo com uma avaliação comparativa, o método de ultrassom pulmonar e a tecnologia de ReDS em pacientes com IC são isolados,^11^ e não há estudos envolvendo a população de pacientes russos.

Assim, o objetivo deste estudo foi uma avaliação comparativa da presença e dinâmica da congestão pulmonar de acordo com a ultrassonografia e a tecnologia de ReDS em pacientes hospitalizados com descompensação aguda de insuficiência cardíaca crônica (ICC).

## Métodos

O estudo incluiu 35 pacientes hospitalizados com ICC no hospital multidisciplinar do Hospital Clínico Estadual VV Vinogradov, Moscou. Usamos amostragem de conveniência. A ICC foi diagnosticada com base nos critérios geralmente aceitos,^6^ com qualquer fração de ejeção do ventrículo esquerdo. O estudo não incluiu pacientes com síndrome coronariana aguda, doenças somáticas e malignas graves, síndrome edematosa de outra etiologia, hepatite aguda com aumento de transaminases >5 limites superiores do normal, imobilização, presença de eletrocardioestimulador, deformidade torácica grave, doenças infecciosas agudas (incluindo pneumonia e COVID-19) e se for impossível realizar análise de bioimpedância da composição corporal (BIVA). Todos os pacientes assinaram um consentimento informado antes dos procedimentos de exame. O estudo foi conduzido de acordo com os padrões de Boas Práticas Clínicas e os princípios da Declaração de Helsinque. O comitê de ética local aprovou o protocolo de pesquisa.

**Figura 1:**
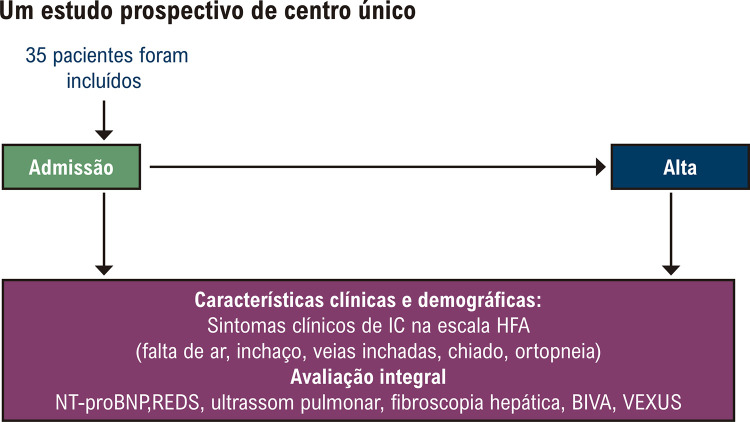
– Desenho do estudo.

Todos os pacientes incluídos no estudo, nas primeiras 24 horas do momento da hospitalização e na alta, foram submetidos a um exame físico, laboratorial e instrumental padrão, incluindo ultrassom pulmonar, NT-proBNP, um estudo usando tecnologia ReDS, fibroelastometria hepática, análise de BIVA, avaliação da congestão venosa de acordo com o protocolo VEXUS. O desenho do estudo é mostrado na Figura 1 .

A escala de avaliação de congestão clínica HFA foi usada para avaliar a congestão clínica.^12^ Cada sintoma e sinal clínico foi avaliado no dia da admissão e alta. A presença de ≥ 1 ponto foi considerada congestão clínica na admissão e estagnação residual com manifestações clínicas na alta.

O NT-proBNP no soro sanguíneo foi determinado pelo ensaio imunoenzimático ELISA usando os sistemas de teste NT-proBNP-ELISA-BEST (Rússia, Vector-Best CJSC).

Ultrassonografia dos pulmões (VIVID iq, GE) com cálculo da soma das linhas B foi realizada em 8 áreas (II e IV m/r entre as linhas paraesternal e médio-clavicular e entre as linhas axilares anterior e média em ambos os lados). 6-15 linhas B foram consideradas congestão leve, 16-30 - moderada e mais de 30 - grave.^13^

**Figura 2:**
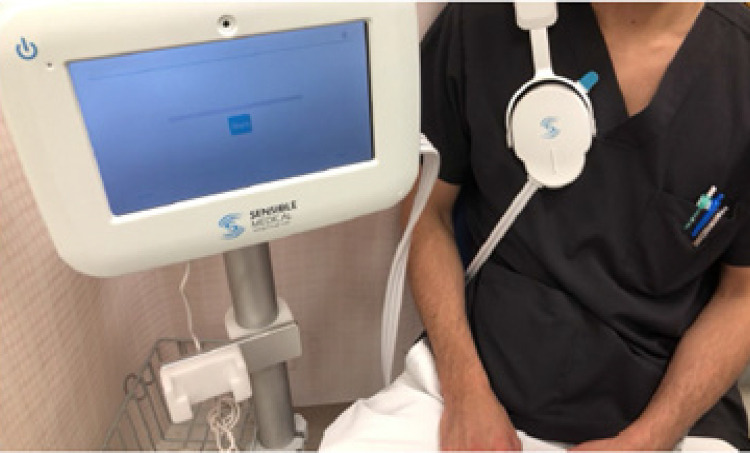
– Tecnologia de sensoriamento dielétrico remoto (ReDS). O dispositivo consiste em um sistema de dois sensores (frontal e traseiro), uma unidade de computação e um monitor.

A tecnologia ReDS é baseada na determinação das propriedades dielétricas do tecido (coeficiente dielétrico): ondas eletromagnéticas de baixa potência passam pelos tecidos do emissor para o receptor; a avaliação das mudanças nos parâmetros das ondas de rádio torna possível medir com precisão o volume total de líquido no tecido, uma vez que a água tem um coeficiente dielétrico muito alto, e os coeficientes dielétricos dos tecidos são determinados principalmente pelo líquido contido nela. Assim, a tecnologia ReDS calcula a relação do volume de líquido para ar e mostra a porcentagem de fluido pulmonar.^10 , 11 , 14^ O estudo foi conduzido de acordo com o protocolo do fabricante. O paciente é equipado com um sensor no lado direito do peito em posição sentada; a medição em si dura 45 segundos ( Figura 2 ). A faixa de valores normais recomendada pelo fabricante é de 20-35%. Se os valores do indicador fossem >35%, o paciente era considerado portador de congestão pulmonar.^15^ A gravidade da congestão foi determinada pelos seguintes valores: 36-40% – grau 1 (aumento do conteúdo de líquido nos pulmões), 41-50% – grau 2 (alto conteúdo de líquido nos pulmões), mais de 50% – grau 3 (alto conteúdo de líquido nos pulmões).

O estudo ReDS foi realizado em cada paciente por dois operadores cegos diferentes, com um intervalo de 20 a 30 minutos, independentemente um do outro, para determinar a variabilidade interoperatória do método.

### Análise estatística

Para o processamento estatístico dos dados, foram utilizados o MedCalc Software’s VAT Versão 19.0 e o SPSS (versão 22.0). Usamos o teste de Shapiro–Wilk para verificar a normalidade dos dados. O teste de Shapiro–Wilk é um método mais apropriado para tamanhos de amostra pequenos (<50 amostras). As variáveis quantitativas foram descritas como a média (M) ± desvio padrão (DP) (com uma distribuição normal) ou como a mediana (Me) e intervalo interquartil (IQR) (com uma distribuição assimétrica). P<0,05 foi considerado significativo. A direção e a força da correlação entre os dois indicadores quantitativos foram estimadas usando o coeficiente de correlação de postos de Spearman.

Para avaliar a variabilidade interoperacional para parâmetros categóricos, foi determinado o coeficiente de concordância, ou kappa de Cohen (κ), que foi calculado usando a fórmula: κ = (po− pe) / (1− pe), onde po é a concordância relativa observada entre os operadores, re é a probabilidade hipotética de uma concordância aleatória (com concordância total κ = 1, e na ausência de consentimento, κ = 0), enquanto: κ = 0-0,2 – baixo nível de consentimento; κ = 0,21-0,4 – nível satisfatório de consentimento; κ = 0,41-0,6 – nível médio de consentimento; κ = 0,61-0,8 – alto nível de consentimento; κ = 0,81-1 – consentimento praticamente total.

## Resultados

As características clínicas e demográficas e os principais indicadores laboratoriais e instrumentais dos pacientes são apresentados na Tabela 1 .

**Tabela 1:** – Características clínicas e demográficas e parâmetros laboratoriais/instrumentais dos pacientes incluídos no estudo (n=35)

Parâmetro	Valor
**Características clínicas e demográficas**	
Gênero (masculino/feminino), n (%)	14 (40%)/21 (60%)
Idade, anos, Me (IQR)	71 [65,5; 78,5]
IMC, kg/m^2^, Ме (IQR)	34,5 [27,0;38,6]
Tabagismo, n (%)	8 (22,9%)
FEVE, % Me (IQR)	52 [40;55]
Hipertensão arterial, n (%)	34 (97,2%)
Anamnese do acidente vascular cerebral, n (%)	5 (14,3%)
Doença cardíaca coronária, n (%)	14 (40,0%)
Anamnese do infarto do miocárdio, n (%)	6 (17,2%)
Fibrilação/flutter atrial, n (%)	22 (62,9%)
Diabetes mellitus tipo 2, n (%)	9 (25,7%)
Doença renal crônica, n (%)	22 (62,9%)
DPOC/AB, n (%)	5 (14,3%)
PAS, MM. pT. cT. Me (IQR)	133 [120,5;146]
PAD, MM. pT. cT. Me (IQR)	80 [70;84,5]
HR, em min. Me (IQR)	85 [74;120]
**Características laboratoriais e instrumentais na internação**	
Densidade hepática, кPа, Me (IQR)	13 [6;21]
Linhas B na ultrassonografia pulmonar, Me (IQR)	8 [4;16]
BIVA, resistência ativa, Оm/m, M ± DP	394 ± 99
BIVA, resistência reativa, Оm/m, Me (IQR)	38 [31;45]
Tamanho da veia cava inferior, mm, M ± DP	22 ± 5
Grau de congestionamento (GRADE) segundo VExUS, n (%)	NOTA 0: 14 (40%)
	GRAU 1: 3 (8,6%)
	2ª SÉRIE: 6 (17,1%)
	3ª SÉRIE: 12 (34,3%)
NT-proBNP, pg/ml, Me (IQR)	1379 (470; 4277)
ReDS, M ± DP	37 ± 6

IMC: índice de massa corporal; FEVE: fração de ejeção do ventrículo esquerdo; DPOC: doença pulmonar obstrutiva crônica; AB: asma brônquica; PAS: pressão arterial sistólica; PAD: pressão arterial diastólica; FC: frequência cardíaca; BIVA: bioimpedância da composição corporal; ReDS: sensoriamento dielétrico remoto.

**Figura 3:**
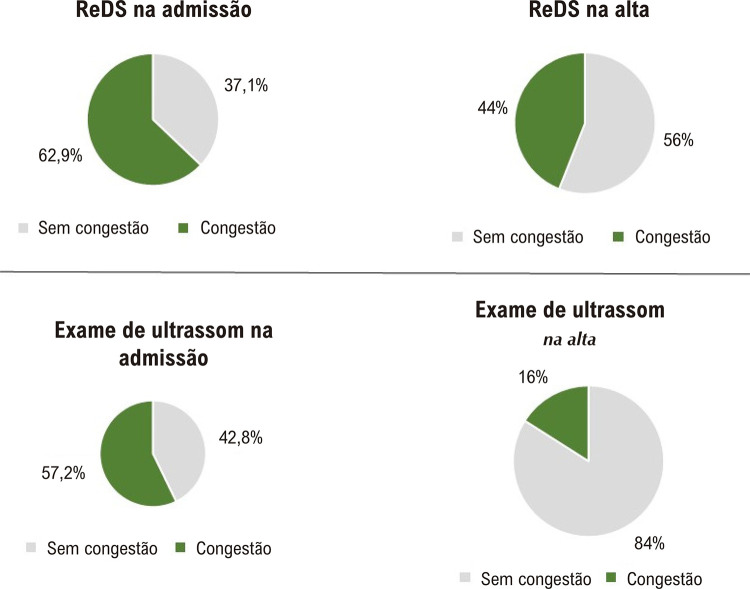
– Frequência de congestão pulmonar em pacientes com TDAH na admissão de acordo com a tecnologia ReDS e ultrassonografia pulmonar (n=35).

A frequência de consentimento para a presença ou ausência de sinais de congestão segundo ambos os métodos na admissão foi de 77,1% (p=0,004) ( Figuras 3 e 4 ), com valor moderado do coeficiente de consentimento kappa Cohen (k =0,53). Na alta, a frequência de concordância entre os métodos foi de 41,7% (p=0,223), e o coeficiente de concordância foi negativo. Ao considerar o hidrotórax como sinal de congestão, além de levar em consideração as linhas B na ultrassonografia pulmonar na admissão, a concordância entre os métodos foi de 71,4% (p=0,033), e o coeficiente de concordância k =0,388. Na alta, a contabilização do hidrotórax não alterou a frequência de concordância entre os dois métodos na detecção de congestão.

**Figura 4:**
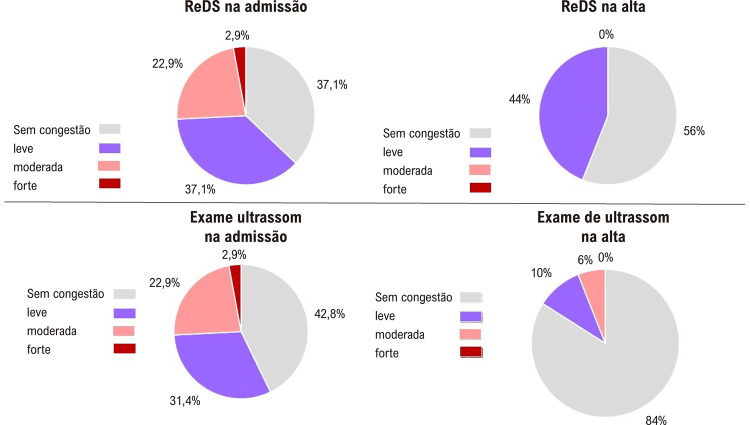
– Frequência de congestão pulmonar em pacientes com TDAH na alta hospitalar segundo a tecnologia ReDS e ultrassonografia pulmonar (n=35).

**Figura 5:**
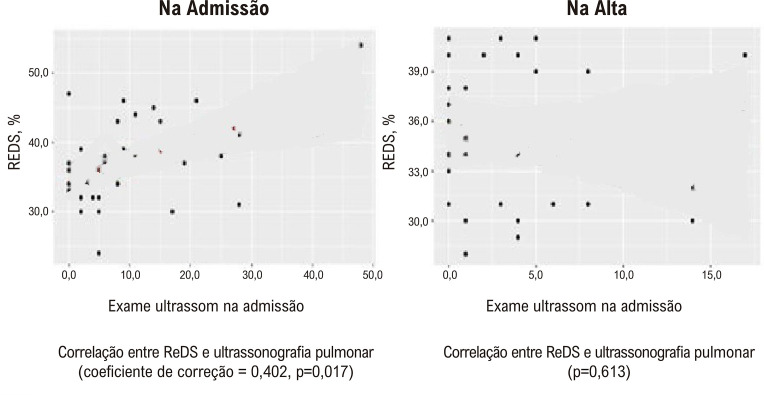
– Relação de correlação entre ReDS (%) e ultrassonografia pulmonar (soma das linhas B).

A variabilidade interoperatória média para o estudo ReDS foi revelada (o coeficiente de variabilidade é de 9,9%). Ao mesmo tempo, a variabilidade entre operadores no momento da hospitalização dos pacientes foi de 12,7% para ReDS na admissão e 6,6% na alta. Para ReDS, o coeficiente de concordância para o estudo ReDS entre operadores para detecção de estagnação foi κ = 0,82 (κ = 0,908 na admissão e κ = 0,657 na alta). Uma correlação moderada foi encontrada entre ReDS (%) e ultrassom pulmonar (soma de linhas B) na admissão. Na alta, nenhuma correlação foi encontrada entre os dois métodos ( Figura 5 ).

## Discussão

A precisão do diagnóstico de congestão pulmonar usando ultrassom dos pulmões é alta; a sensibilidade e especificidade deste método excedem 95%. O consenso de especialistas recomenda ultrassom dos pulmões para o diagnóstico de congestão pulmonar.^16^ Portanto, o ultrassom pulmonar neste estudo foi considerado o “padrão ouro” para avaliar a congestão pulmonar. No entanto, é um método semiquantitativo e requer equipamento apropriado e especialistas altamente qualificados.

Como um método alternativo para quantificar o grau de congestão nos pulmões e exibir a porcentagem de fluido pulmonar em 45 segundos, o sistema ReDS pode ser usado. Esta é uma tecnologia não invasiva que não requer nenhuma técnica especializada.

Foi demonstrada uma forte correlação entre ReDS e a dinâmica da congestão pulmonar no contexto da terapia diurética durante a hospitalização de pacientes com TDAH.^17^ Foi demonstrado que os valores de ReDS têm uma forte correlação com outros métodos de avaliação, incluindo tomografia computadorizada de alta resolução (0,90 (IC 95% 0,85-0,95)^18^ e cateterização do coração direito.^19^ Em um estudo realizado no Japão, houve uma correlação moderada entre os valores de ReDS e a porcentagem de área de alta atenuação na tomografia computadorizada (r = 0,65, p < 0,001). Além disso, foi demonstrado que o valor de ReDS é um preditor independente de congestão pulmonar após correção para o valor do peptídeo natriurético (NT-proBNP) e o peso corporal do paciente.^20^

O cateterismo do coração direito é o padrão ouro para avaliar a gravidade da congestão pulmonar por meio da medição da pressão capilar pulmonar (PCP). No entanto, o cateterismo do coração direito é invasivo, doloroso e associado ao risco de exacerbação da IC, especialmente em pacientes com hemodinâmica instável, bem como naqueles que recebem anticoagulantes. Em um estudo conduzido em Israel em 139 pacientes com IC, foi encontrado um coeficiente de correlação positivo entre os valores de ReDS e pressão da artéria pulmonar (r = 0,492, p < 0,001), bem como os valores de ReDS e pressão venosa central (r = 0,406, p < 0,001). Foi demonstrado que o valor de ReDS (valor limite de 34%) tem alta sensibilidade (90,7%), especificidade (77,1%) e valor prognóstico negativo (94,9%) para determinar a PCP de 18 mmHg.^21^ Em outro estudo, foi encontrada uma correlação moderada entre os valores de ReDS e PCP (r = 0,698, p < 0,001), e o valor de REDS 28% indica um valor limite para prever PCP > 15 mmHg com sensibilidade (0,70) e especificidade (0,75) suficientemente altas.^16^

Em nosso estudo, a congestão pulmonar na admissão, de acordo com o ReDS, foi diagnosticada em 62,9%, de acordo com o ultrassom, em 57,2% dos pacientes. Foi encontrada uma correlação moderada entre o ReDS (%) e o ultrassom pulmonar (soma das linhas B) na admissão (coeficiente de correlação de Spearman = 0,402; p = 0,017). A variabilidade interoperacional dos valores do REDS também foi estudada. O coeficiente de concordância para o estudo ReDS entre operadores para detecção de estagnação foi κ = 0,82 (κ = 0,908 na admissão e κ = 0,657 na alta), o que indica concordância quase completa nos valores entre os dois operadores. Os dados da literatura confirmam isso.

Um estudo no Japão envolvendo 10 voluntários saudáveis também demonstrou confiabilidade muito alta das medições de ReDS entre três operadores (0,966, IC de 95%: 0,952-0,976), o que sugere que uma única medição de ReDS é confiável.^22^

### Limitações do estudo

O número de pacientes incluídos no estudo foi pequeno. No futuro, planejamos estudar o significado prognóstico de longo prazo da congestão pulmonar subclínica em pacientes com ICAD nesta população de pacientes.

## Conclusão

Assim, a tecnologia de exame dielétrico remoto (ReDS) tem correlação moderada com a ultrassonografia pulmonar em relação à avaliação da congestão pulmonar em pacientes hospitalizados com descompensação aguda de ICC. No entanto, deve-se dizer que atualmente esses métodos podem ser considerados complementares, e o uso da tecnologia ReDS em pacientes com IC requer mais estudos.
