# Supplementary material for: Avaliação da Congestão Pulmonar por Ultrassom e Sensoriamento Dielétrico Remoto (ReDS) em Pacientes Hospitalizados com Insuficiência Cardíaca
Source: Arq Bras Cardiol. 2024 Oct 23;121(10):e20240128. [Article in Portuguese] doi: 10.36660/abc.20240128 (PMC11634205; doi:10.36660/abc.20240128)
Supplement: Translation

# Translation: Assessment of Pulmonary Congestion According to Ultrasound and Remote Dielectric Sensing (ReDS) in Patients Hospitalized With Heart Failure

**DOI:** 10.36660/abc.20240128i

**Authors:** Zhanna Kobalava, Ayten Fuadovna Safarova, Veronika Tolkacheva, Flora Elisa Cabello-Montoya, Olga Tairovna Zorya, Ivan Sergeevich Nazarov, Artem Alekseevich Lapshin, Ilya Pavlovich Smirnov, Nutsiko Ivanovna Khutsishvili, Maria Vatsik-Gorodetskaya

**Affiliations:** 1 Russia RUDN University, Moskva, Moskovskaa Oblast – Russia

**Keywords:** Heart Failure, Pulmonary Edema, Ultrasonography

## Abstract

**Background:**

The reduction of pulmonary congestion is an essential clinical target in the management of chronic heart failure. The remote dielectric sensing (ReDS) system is a recently introduced non-invasive technology used to easily estimate the degree of lung fluid volume without any expert techniques.

**Objective:**

To conduct a comparative assessment of pulmonary congestion according to ultrasound and ReDS technology in patients hospitalized with decompensation of chronic heart failure (CHF)

**Methods:**

The pilot single-center study included patients hospitalized with CHF decompensation. On admission and upon discharge, lung ultrasound and ReDS technology were simultaneously performed. Ultrasound of the lungs was performed according to the protocol with an assessment of 8 zones and calculation of the sum of B-lines. Pulmonary congestion was confirmed with a sum of B-lines ≥5, ReDS congestion if >35%. A p<0.05 was considered statistically significant.

**Results:**

35 patients were included in the study; 40% (n=14) were men, the average age was 71 (65.5; 78.5) years. Pulmonary congestion, according to ultrasound, was 57.1% (n=20), and according to ReDS, 62,9% (n=22). A moderate correlation was found between ReDS (%) and lung ultrasound (sum of B-lines) upon admission (Spearman correlation coefficient = 0.402; p=0.017). There was no correlation between the two methods at discharge (p=0.613).

**Conclusion:**

There was a moderate correlation between ReDS and lung ultrasound in relation to the detection of pulmonary congestion at admission.

## Introduction

Acute decompensation of heart failure (ADHF) is based on a multilevel cascade of pathological reactions, which include hemodynamic overload and venous congestion. Assessing the volemic status or hydration status and, if possible, quantifying the degree of congestion is one of the most important tasks in the management strategy of patients in both inpatient and outpatient settings.

Residual congestion at discharge is a serious problem associated with an increased risk of re-hospitalization and mortality.^1 - 3^In addition, patients without clinical manifestations of congestion may experience subclinical congestion at discharge, which is detected only by laboratory and instrumental methods, and clinical manifestations may develop even before the end of the first week of observation of the patient on an outpatient basis.^2 , 3^

More than 90% of HF-related hospitalizations occur due to the presence of pulmonary congestion (CHAMPION trial).^4^Early detection of pulmonary congestion is extremely important, as it allows to prevent the development of a decompensation episode in time, correct treatment in time and improve the prognosis.

Currently, it is recommended to use chest X-ray, lung ultrasound, echocardiography and/or natriuretic peptides to detect congestion in patients with acute HF.^5 , 6^When analyzing data from large foreign registries and randomized clinical trials, where lung ultrasound and radiography were compared for the diagnosis of cardiogenic interstitial syndrome in patients with HF, it was found that lung ultrasound is not only a more sensitive method for assessing pulmonary congestion but also has independent prognostic value.^7 - 9^

However, none of the methods make it possible to assess the degree of fluid overload accurately, and therefore, there remains a need to find a new, accurate, and simple technology for assessing congestion in the lungs. The urgency of this problem has led to the development of a new non-invasive Remote Dielectric Sensing technology (ReDS), which is a validated quantitative method for measuring the total volume of fluid in the lungs by determining the dielectric properties of tissue. The use of this technology makes it possible to quickly, noninvasively, and quantitatively measure the fluid content in the lungs, makes it possible to optimize the treatment regimen and reduces the number of repeated hospitalizations.^10^However, according to a comparative assessment, the lung ultrasound method and remote dielectric sensing technology (ReDS) in patients with HF are isolated,^11^and there are no studies involving the Russian patient population.

Thus, the purpose of this study was a comparative assessment of the presence and dynamics of pulmonary congestion according to ultrasound and remote dielectric sensing technology (ReDS) in patients hospitalized with acute decompensation of chronic heart failure (ACHF).

## Methods

The study included 35 patients hospitalized with ACHF in the multidisciplinary hospital of the V.V. Vinogradov State Clinical Hospital, Moscow. We use convenience sampling. ACHF was diagnosed based on generally accepted criteria,^6^with any left ventricular ejection fraction. The study did not include patients with acute coronary syndrome, severe somatic and malignant diseases, edematous syndrome of other etiology, acute hepatitis with increased transaminases >5 upper limits of normal, immobilization, the presence of an electrocardiostimulator, severe chest deformity, acute infectious diseases (including pneumonia and COVID-19), and if it is impossible to perform bioimpedance analysis of body composition (BIVA). All patients signed an informed consent prior to the examination procedures. The study was conducted in accordance with the standards of Good Clinical Practice and the principles of the Helsinki Declaration. The local ethics committee approved the research protocol.

All patients included in the study, in the first 24 hours from the moment of hospitalization and upon discharge, underwent a standard physical, laboratory, and instrumental examination, including lung ultrasound, NT-proBNP, a study using ReDS technology, liver fibroelastometry, bioimpedance analysis of body composition, assessment of venous congestion according to the VEXUS protocol. The design of the study is shown in Figure 1 .

**Figure 1:** – Design of the study.

The HFA clinical congestion assessment scale was used to assess clinical congestion.^12^Each clinical symptom and sign was evaluated on the day of admission and discharge. The presence of ≥ 1 point was considered clinical congestion at admission and residual stagnation with clinical manifestations at discharge.

NT-proBNP in blood serum was determined by ELISA enzyme immunoassay using the NT-proBNP-ELISA-BEST test systems (Russia, Vector-Best CJSC).

Ultrasound of the lungs (VIVID iq, GE) with the calculation of the sum of B-lines was performed in 8 areas (II and IV m/r between the parasternal and midclavicular lines and between the anterior and middle axillary lines on both sides). 6- 15 B-lines were regarded as mild congestion, 16-30 - moderate, and more than 30 – severe.^13^

The ReDS technology is based on the determination of the dielectric properties of tissue (dielectric coefficient): low-power electromagnetic waves pass through tissues from the emitter to the receiver; the assessment of changes in radio wave parameters makes it possible to accurately measure the total volume of liquid in the tissue, since water has a very high dielectric coefficient, and the dielectric coefficients of tissues are determined mainly by the liquid contained in it. Thus, the ReDS technology calculates the volume ratio of liquid to air and shows the percentage of pulmonary fluid.^10 , 11 , 14^The study was conducted according to the manufacturer’s protocol. The patient is fitted with a sensor on the right side of the chest in a sitting position; the measurement itself lasts 45 seconds ( Figure 2 ). The manufacturer’s recommended range of normal values is 20-35%. If the values of the indicator were >35%, then the patient was considered to have pulmonary congestion.^15^The severity of congestion was determined by the following values: 36-40% – grade 1 (increased fluid content in the lungs), 41-50% - grade 2 (high fluid content in the lungs), more than 50% - grade 3 (extremely high fluid content in the lungs).

**Figure 2:** – Remote dielectric sensing technology (ReDS). The device consists of a system of two sensors (front and rear), a computing unit, and a monitor.

The ReDS study was performed on each patient by 2 different blinded operators with an interval of 20-30 minutes independently of each other to determine the interoperative variability of the method.

### Statistical analysis

For statistical data processing, MedCalc Software’s VAT Version 19.0 and SPSS (version 22.0) were used. We use the Shapiro–Wilk test to verify the normality of the data. The Shapiro–Wilk test is a more appropriate method for small sample sizes (<50 samples). Quantitative variables were described as the mean (M) ± standard deviation (SD) (with a normal distribution) or as the median (Me) and interquartile range (IQR) (with an asymmetric distribution). P<0.05 was considered significant. The direction and strength of the correlation between the two quantitative indicators were estimated using Spearman’s rank correlation coefficient.

To assess interoperational variability for categorical parameters, the coefficient of agreement, or Cohen’s kappa (κ), was determined, which was calculated using the formula: κ = (po− pe) / (1− pe), where po is the relative observed agreement between operators, re is the hypothetical probability of a random agreement (with full agreement κ = 1, and in the absence of consent, κ = 0), while: κ = 0-0.2 – low level of consent; κ = 0.21-0.4 – satisfactory level of consent; κ = 0.41-0.6 – average level of consent; κ = 0.61-0.8 – high level of consent; κ = 0.81-1 – practically full consent.

## Results

The clinical and demographic characteristics and the main laboratory and instrumental indicators of patients are presented in Table 1 .

**Table 1:** – Clinical and demographic characteristics and laboratory/instrumental parameters of patients included in the study (n=35)

Parameter	Value
**Clinical and demographic characteristics**	
Gender (male/female), n (%)	14 (40%)/21 (60%)
Age, years, Ме (IQR)	71 [65.5; 78.5]
BMI, kg/m^2^, Ме (IQR)	34.5 [27.0;38.6]
Smoking, n (%)	8 (22.9%)
LVEF, % Ме (IQR)	52 [40;55]
Arterial hypertension, n (%)	34 (97.2%)
Anamnesis of stroke, n (%)	5 (14.3%)
Coronary heart disease, n (%)	14 (40.0%)
Anamnesis of myocardial infarction, n (%)	6 (17.2%)
Atrial fibrillation/flutter, n (%)	22 (62.9%)
Diabetes mellitus 2 type, n (%)	9 (25.7%)
Chronic kidney disease, n (%)	22 (62.9%)
COPD/BA, n (%)	5 (14.3%)
SBP, мм. рт. ст. Ме (IQR)	133 [120.5;146]
DBP, мм. рт. ст. Ме (IQR)	80 [70;84.5]
HR, in min. Ме (IQR)	85 [74;120]
**Laboratory and instrumental characteristics at hospitalization**	
Liver density, кPа, Ме (IQR)	13 [6;21]
B-lines in lung ultrasound, Ме (IQR)	8 [4;16]
BIVA, active resistance, Оm/m, M ± SD	394 ± 99
BIVA, reactive resistance, Оm/m, Ме (IQR)	38 [31;45]
The size of the inferior vena cava, mm, M ± SD	22 ± 5
The degree of congestion (GRADE) according to VExUS, n (%)	GRADE 0: 14 (40%)
	GRADE 1: 3 (8.6%)
	GRADE 2: 6 (17.1%)
	GRADE 3: 12 (34.3%)
NT-proBNP, pg/ml, Ме (IQR)	1379 (470; 4277)
ReDS, M ± SD	37 ± 6

BMI: body mass index; LVEF: left ventricle ejection fraction; COPD: chronic obstructive pulmonary disease; BA: bronchial asthma; SBP: systolic blood pressure; DBP: diastolic blood pressure; HR: heart rate; BIVA: bioimpedance analysis of body composition; ReDS: remote dielectric sensing.

The frequency of consent for the presence or absence of signs of congestion according to both methods at admission was 77.1% (p=0.004) ( Figures 3 and 4 ), with a moderate value of the kappa Cohen consent coefficient (k =0.53). At discharge, the frequency of agreement between the methods was 41.7% (p=0.223), and the coefficient of agreement was negative. When taking into account hydrothorax as a sign of congestion, in addition to taking into account B-lines on lung ultrasound at admission, the agreement between the methods was 71.4% (p=0.033), and the coefficient of agreement k =0.388. At discharge, hydrothorax accounting did not change the frequency of agreement between the two methods in detecting congestion.

**Figure 3:** – The frequency of pulmonary congestion in patients with ADCHD at admission according to the ReDS technology and lung ultrasound (n=35).

**Figure 4:** – The frequency of pulmonary congestion in patients with ADCHD at discharge according to the ReDS technology and lung ultrasound (n=35).

The average interoperative variability for the ReDS study was revealed (the coefficient of variability is 9.9%). At the same time, the variability between operators at the time of hospitalization of patients was 12.7% for ReDS upon admission and 6.6% upon discharge. For ReDS, the coefficient of agreement for the ReDS study between operators for detecting stagnation was κ =0.82 (κ =0.908 at admission and κ =0.657 at discharge). A moderate correlation was found between ReDS (%) and lung ultrasound (sum of B-lines) upon admission. At discharge, no correlation was found between the two methods ( Figure 5 ).

**Figure 5:** – Correlation relationship between ReDS (%) and lung ultrasound (sum of B-lines).

## Discussion

The accuracy of diagnosis of pulmonary congestion using ultrasound of the lungs is high; the sensitivity and specificity of this method exceed 95%. The consensus of experts recommends ultrasound of the lungs for the diagnosis of pulmonary congestion.^16^Therefore, lung ultrasound in this study was considered the “gold standard” for assessing pulmonary congestion. However, it is a semi-quantitative method and requires appropriate equipment and highly qualified specialists.

As an alternative method for quantifying the degree of congestion in the lungs and displaying the percentage of pulmonary fluid in 45 seconds, the ReDS system can be used. This is a non-invasive technology that does not require any expert techniques.

A strong correlation was demonstrated between ReDS and the dynamics of pulmonary congestion on the background of diuretic therapy during hospitalization of patients with ADHD.^17^It has been shown that the ReDS values have a strong correlation with other assessment methods, including high-resolution computed tomography (0.90 (95% CI 0.85-0.95)^18^and catheterization of the right heart.^19^In a study conducted in Japan, there was a moderate correlation between ReDS values and the percentage of high attenuation area in computed tomography (r = 0.65, p <0.001). In addition, it was shown that the ReDS value is an independent predictor of pulmonary congestion after correction for the value of natriuretic peptide (NT-proBNP) and the patient’s body weight.^20^

Catheterization of the right heart is the gold standard for assessing the severity of pulmonary congestion by measuring the pulmonary capillary wedge pressure (PCWP). However, catheterization of the right heart is invasive, painful, and associated with the risk of exacerbation of heart failure, especially in patients with unstable hemodynamics, as well as in those who receive anticoagulants. In a study conducted in Israel in 139 patients with HF, a positive correlation coefficient was found between the values of ReDS and pulmonary artery pressure (r=0.492, p<0.001), as well as the values of both ReDS and central venous pressure (r=0.406, p<0.001). It has been shown that the ReDS value (threshold value of 34%) has high sensitivity (90.7%), specificity (77.1%), and negative prognostic value (94.9%) for determining the PCWP of 18 mmHg.^21^In another study, a moderate correlation was found between the values of ReDS and PCWP (r=0.698, p<0.001), and the value of REDS 28% indicates a threshold value for predicting PCWP >15 mmHg with sufficiently high sensitivity (0.70) and specificity (0.75).^16^

In our study, lung congestion at admission, according to ReDS, was diagnosed in 62.9%, according to ultrasound, in 57.2% of patients. A moderate correlation was found between ReDS (%) and lung ultrasound (sum of B-lines) upon admission (Spearman correlation coefficient = 0.402; p=0.017). The interoperational variability of REDS values was also studied. The coefficient of agreement for the ReDS study between operators for detecting stagnation was κ =0.82 (κ =0.908 at admission and κ =0.657 at discharge), which indicates almost complete agreement in values between the two operators. The literature data confirms this.

A study in Japan involving 10 healthy volunteers also demonstrated very high reliability of ReDS measurements between three operators (0.966, 95% CI: 0.952- 0.976), which suggests that a single measurement of ReDS is reliable.^22^

### Limitations of the study

The number of patients included in the study was small. In the future, we plan to study the long-term prognostic significance of subclinical lung congestion in patients with ADHF in this patient population.

## Conclusion

Thus, the technology of remote dielectric examination (ReDS) has a moderate correlation with lung ultrasound in relation to the assessment of pulmonary congestion in patients hospitalized with acute decompensation of chronic heart failure. However, it should be said that currently these methods can be considered complementary, and the use of ReDS technology in patients with HF requires further study.
